# Characterizing technical success and clinical outcomes in patients with pulmonary embolism treated with ultrasound-assisted catheter-directed thrombolysis (USAT): a retrospective, single-center cohort study

**DOI:** 10.1007/s00392-025-02643-2

**Published:** 2025-05-19

**Authors:** Marius Wessinger, Nadine Gauchel, Daniel Strobel, Dawid L. Staudacher, Tobias Wengenmayer, Constantin von zur Mühlen, Hans-Jörg Busch, Katrin Fink, Katharina Müller-Peltzer, Fabian Bamberg, Klaus Kaier, Dirk Westermann, Christoph B. Olivier

**Affiliations:** 1 Department of Cardiology and Angiology, University Heart Center Freiburg-Bad Krozingen, Medical Center - University of Freiburg, Faculty of Medicine, University of Freiburg, Freiburg, Germany; 2 Interdisciplinary Medical Intensive Care, Medical Center - University of Freiburg, Faculty of Medicine, University of Freiburg, Freiburg, Germany; 3 Department of Emergency Medicine, University Hospital of Freiburg, Faculty of Medicine, University of Freiburg, Freiburg, Germany; 4 Department of Radiology, Medical Center - University of Freiburg, Faculty of Medicine, University of Freiburg, Freiburg, Germany; 5 Institute for Medical Biometry and Statistics, Medical Center - University of Freiburg, Faculty of Medicine, University of Freiburg, Freiburg, Germany

**Keywords:** Pulmonary embolism, Catheter-directed thrombolysis, Right ventricular dysfunction, Ultrasound-assisted catheter-directed thrombolysis

## Abstract

**Background:**

Ultrasound-assisted catheter-directed thrombolysis (USAT) is a treatment option for patients with intermediate–high- or high-risk pulmonary embolism (PE). This study aimed to describe the use of USAT and its clinical outcomes.

**Methods:**

In this single-center retrospective cohort study, all USAT procedures performed between May 2019 and June 2022 were included. Data were collected from electronic health records. The primary outcome was reduction in right vs. left ventricular diameter (RV/LV ratio). Secondary outcomes were in-hospital mortality and bleeding.

**Results:**

A total of 107 patients underwent USAT for PE. The median age was 64 (IQR 53–75) years and 59% were male. Technical success of USAT was achieved in 105 (98%) cases. In 32 cases data on RV/LV ratio changes were available. RV/LV ratio decreased by 0.29 ± 0.19 from 1.19 (1.02–1.35) to 0.89 (0.78–1.00). 12 (11%) patients had a fatal outcome. Bleeding complications were observed in 28 (26%) patients, including 14 (13%) major bleedings and 0 (0%) fatal. Both, death and bleeding rates were significantly higher in high-risk patients.

**Conclusion:**

We observed a high technical success of USAT in patients with intermediate–high- and high-risk pulmonary embolism, along with a significant early reduction of RV/LV ratio following treatment.

**Graphical abstract:**

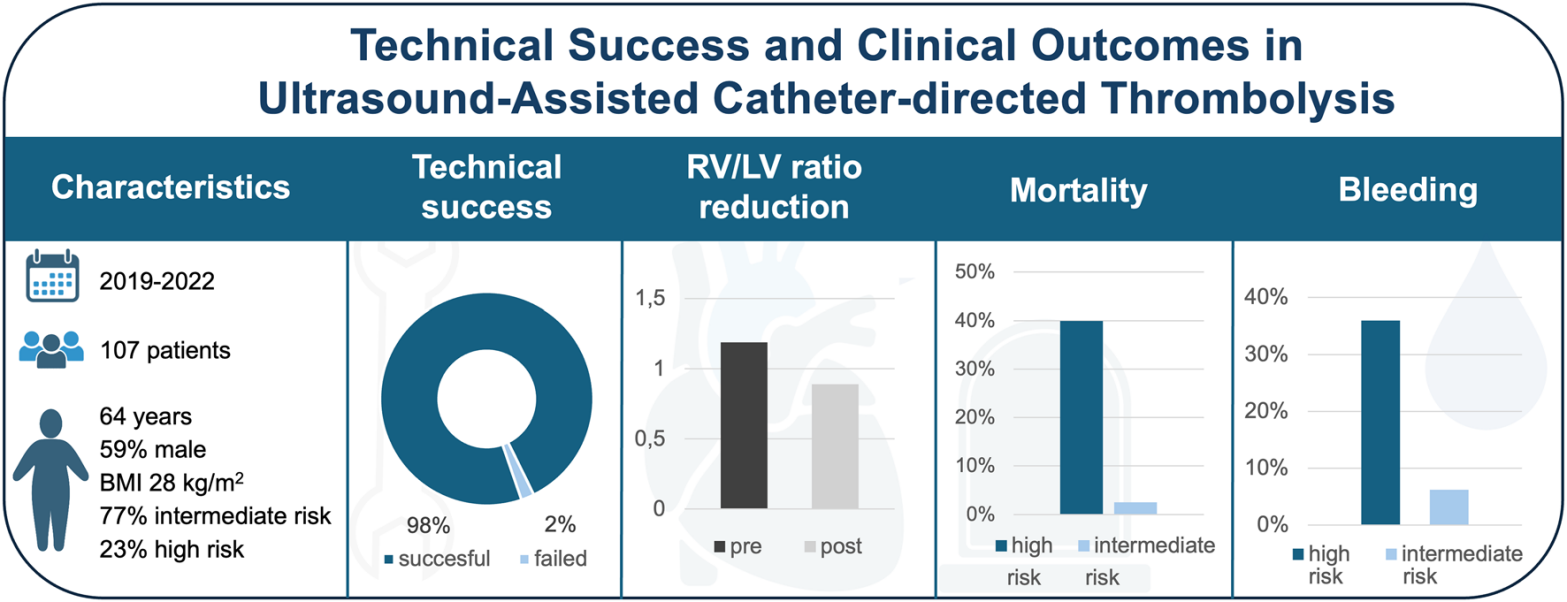

**Supplementary Information:**

The online version contains supplementary material available at 10.1007/s00392-025-02643-2.

## Introduction

Pulmonary embolism (PE) is an acute cardiovascular syndrome associated with a high mortality, particularly in older patients with comorbidities [1]. Implementing structured diagnosis pathways and emphasizing rigorous anticoagulation has led to a reduction of mortality and improved clinical outcomes [2]. In addition, upcoming invasive therapeutic procedures are promising options for critically ill patients. Especially in the group of intermediate–high-risk PE patients, treatment options include anticoagulation, fibrinolysis, catheter-directed therapy (CDT), and/or surgical thrombectomy. However, the best use of catheter-directed therapy remains uncertain [2]. Guidelines recommend anticoagulation for patients with intermediate-risk PE and in case of deterioration interventional thrombus reduction without favoring a specific procedure [3]. In clinical practice, the use of CDT has increased over the past years and several randomized controlled trials (RCT) are currently evaluating the advantages and disadvantages of different methods [4]. The optimal patient selection remains challenging. This single-center cohort study provides results about the patients undergoing ultrasound-assisted catheter-directed thrombolysis at one institution with focus on technical success, patient characteristics, and clinical outcomes.

## Methods

### Study design

In this retrospective, observational, single-center cohort study at the Department of Cardiology and Angiology, University Heart Center, University of Freiburg, we included all patients who underwent ultrasound-assisted catheter-directed thrombolysis (USAT) at our clinic between May 2019 and June 2022 with pulmonary embolism. The protocol and amendments were approved by the local ethics committee, privacy and security offices, and institutional review board (22–1083-retro). Patients were identified via the official OPS key 8–838.70, used for USAT procedures. 109 procedures were identified according to operations and procedure keys. Inclusion and exclusion criteria are listed in supplemental Table S1. Due to an expanded access, two patients were excluded as their treatment differed significantly from the standard operating procedure (SOP). One patient suffered from thrombus formation on a pacemaker probe. In one case, no local lysis was administered. Thus, data from 107 patients were included for analysis. The decision for USAT was based on international guidelines regarding medical history, clinical conditions, and laboratory markers. Right ventricular dysfunction was assessed in echocardiography and computed tomography. The team included an experienced interventional cardiologist and a specialist for intensive care and emergency medicine. The final treatment decisions were were taken by the treating physician, which may have led to biases and resulted in a heterogenous group of patients.

### Risk stratification

The assignment to intermediate or high-risk was taken from the medical record, or calculated and verified, based on sPESI, RV dysfunction, and troponin, if data was available. If no image data was available, the statement about RV dysfunction described in the reports was adopted. In two cases, risk stratification as stated in the record differed after verification. In these cases, the higher category was used.

### Intervention

After interdisciplinary treatment decision, USAT was performed by an interventional, board-certified cardiologist. Ultrasound-guided venous access was not mandatory and left at the discretion of the interventionalist. The standard access site was the right femoral vein. After catheter placement in the proper position, a bolus of 0.5–1 mg alteplase was administered per catheter, followed by a continuous administration of 1 mg alteplase per hour for at least 6 h. If there were no signs of bleeding, a total of 10 h of administration with a total of 20 mg (10 mg/catheter) alteplase was completed. Intravenous anticoagulation was continued with a general target partial thromboplastin time (PTT) of 60–80 s. Postprocedural monitoring in an intensive care unit was mandatory for the standard operating procedure at least while intravenous lysis was administered. After completion of local lysis, both ultrasound catheters and the venous sheaths were removed, and a pressure bandage was applied for at least 6 h.

### Outcomes and follow-up

#### Technical success

A procedure was defined as technically successful, if several criteria were met. First, venous puncture and guidewire placement were achieved. Second, catheter placement was executed as intended, considering the necessity of unilateral or bilateral treatment. Third, a successful application of a bolus, followed by a continuous application of local lysis was established and no device failure occurred during therapy.

#### Clinical outcomes

**Fig. 1:**
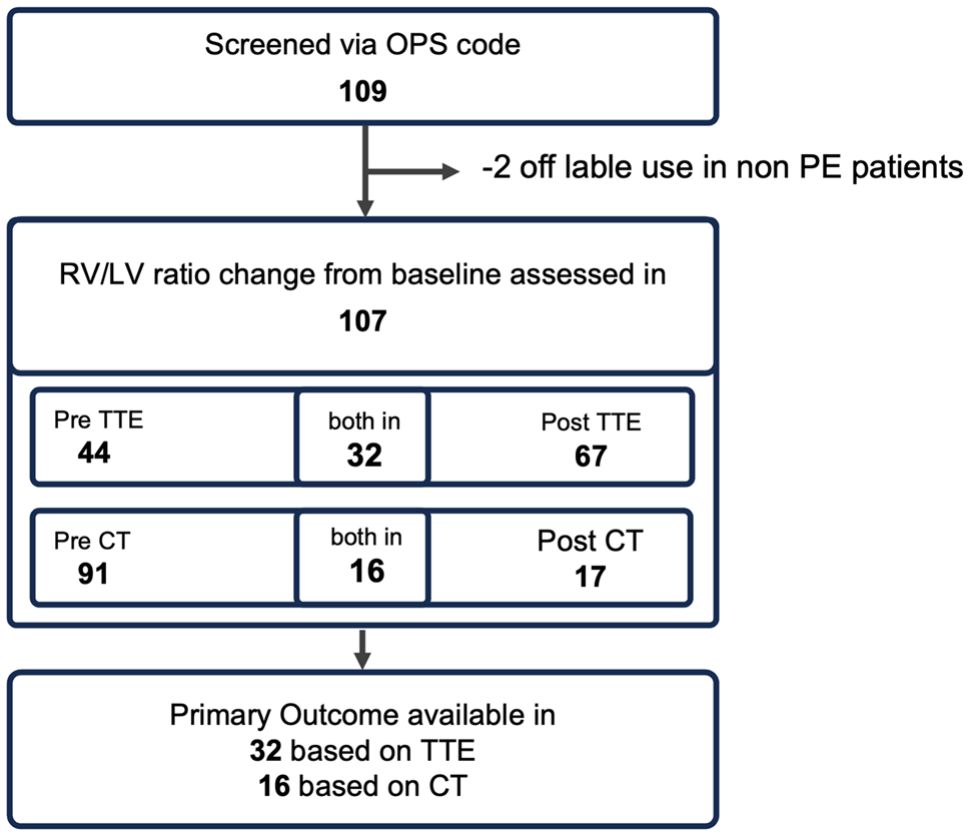
Inclusion Flowchart. After screening, 109 USAT cases were identified by OPS code, 2 cases were excluded because of use in non-PE patients. Of those 107 cases, in 44 cases TTE imaging before and in 67 after EKOS was available. RV/LV ratio change was calculated in 32 cases. Based on CT images, RV/LV ratio was calculated in 16 cases. Image data before EKOS was available in 91 cases, after EKOS in 17 cases

The primary clinical outcome was the change in the right to left ventricular diameter ratio as an indication for improved right ventricular function [5, 6]. Both CT scans and TTE image data were analyzed to obtain the change in RV/LV ratio (Fig. 1). Secondary outcomes were intrahospital death and bleeding. End-diastolic right and left ventricular diameter was measured in apical four-chamber view at the maximal transverse diameter in the basal one-third of the RV and adjusted by two independent investigators [7]. The ISTH classification was used for bleeding assessment [8].

### Statistical considerations

For statistical analysis, paired and unpaired *t*-tests were used. The Kolmogorov–Smirnov test was used to test for normal distribution. Linear and logistic regression were used to test for correlation. For contingency table analysis, Fisher’s exact test was used. Results were combined in forest plots showing odds ratio and 95% confidence interval. Statistical significance was reached with *p* < 0.05.

## Results

### Patient population

Of the 107 patients analyzed in this study, 63 (59%) were male. The median age was 64 years (IQR 53–75), with a median weight of 84 kg (78–102). Table 1 summarizes the baseline characteristics. Relevant preconditions were immobilization in 26 (24%), an active malignancy condition in 15 (14%), and previous deep vein thrombosis (DVT) in 28 patients (26%). 18 patients (17%) were under antiplatelet therapy and 9 (8%) were under oral anticoagulation. 80 patients (75%) were classified as intermediate–high and 25 (23%) as high-risk. Two (2%) patients were classified as intermediate–low-risk. Median troponin at baseline was 62.6 ng/l (26.85─128.5), median NT proBNP was 2158 pg/ml (836─4355), and lactate was 1.7 mg/dl (1.2─2.8). Median SpO_2_ at baseline was 86% (72─94) and 4 l/min (2─8) of oxygen was applied.

**Table 1:** Patient characteristics

Baseline characteristics	Total *n* = 107
Demographics	
Male	63 (59%)
Age, years	64 (53–75)
Weight [kg]	84 (78–102)
BMI [kg/m^2^]	27.9 (24.7–32.7)
Medical history at presentation	
Diabetes mellitus	17 (16%)
Atrial fibrillation	2 (2%)
Coronary heart disease	9 (8%)
Chronic heart failure	10 (9%)
Previous pulmonary embolism	16 (15%)
Previous deep vein thrombosis	28 (26%)
Active malignancy	15 (14%)
Immobilization	26 (24%)
Bedriddenness	6 (6%)
Inflammatory bowel disease	6 (6%)
Active autoimmune disease	6 (6%)
Antiphospholipid syndrom	0 (0%)
Anticoagulation	9 (8%)
Antiplatelet therapy	18 (17%)
Pulmonary embolism severity	
sPESI	1 (1─2)
Risk stratification	
High	25 (23%)
Intermediate–high	80 (75%)
Intermediate–low	2 (2%)
Previous systemic lysis	6 (6%)
ECLS/ECMO	10 (9%)
ICU (days)	3 (2–5)
Rescue therapy	1 (1%)

### Ultrasound-assisted catheter-directed thrombolysis procedure

Table 2 shows relevant technical aspects. A proper catheter placement was obtained in 105 (98%) cases. Two catheter placements failed due to massive thrombus formation in pulmonary artery or inferior vena cava. In two cases, a concomitant DVT complicated inguinal puncture. In 94 (88%) patients, bilateral catheters were placed. The median procedural duration was 47 (36–61) min. The treatment was associated with an ICU stay of 3 (2–5) days and a total hospital stay of 6 (4–9) days. Extracorporeal circulation was used in 10 cases (9%) and 19 (18%) were on ventilation. One patient needed an additional rescue therapy in the form of systemic lysis.

**Table 2:** Technical aspects

EKOS	Total *N* = 107
Successful procedure	105 (98%)
Number of catheters	
Unilateral	13 (12%)
Bilateral	94 (88%)
Pulmonary arterial pressure [mmHg]	33 (26–39)
Dose area product [μGym^2^]	535 (268–1203)
Contrast agent [mg]	10 (0–20)
Total duration [min]	47 (36–61)
Bolus administered	107 (100%)
Lysis	
Rate [mg/h]	1 (1–1)
Dose of lysis [mg]	20 (20–20)
Duration of EKOS-Lysis [h]	10 (10–10)

### Primary outcomes

**Fig. 2:**
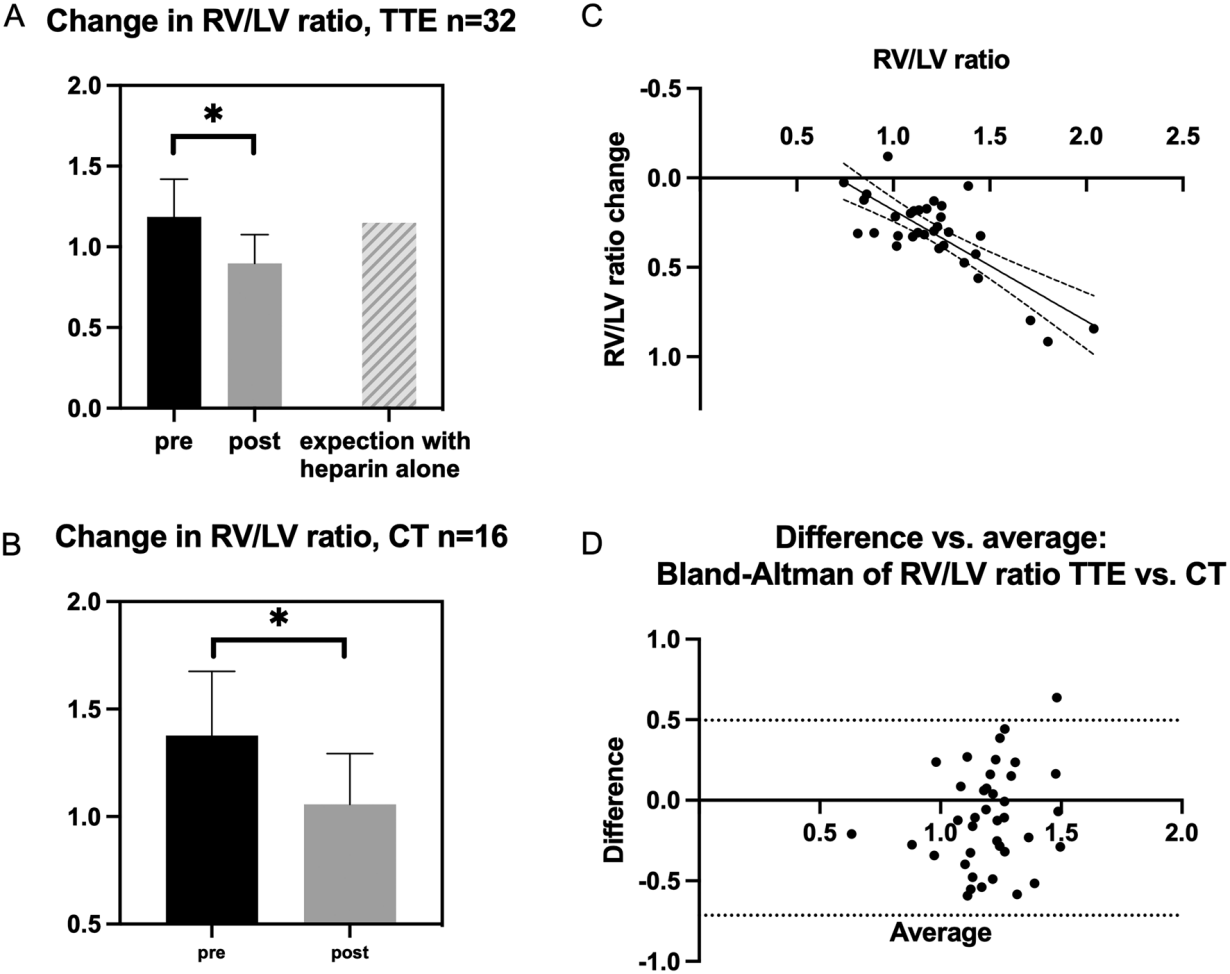
Changes in RV/LV ratio. **A** RV/LV ratio was significantly reduced based on TTE imaging. **B** A significant reduction of RV/LV ratio was shown in CT image analysis. **C** Reduction in RV/LV ratio correlates significantly with RV/LV ratio at baseline. **D** Bland Altman plot comparing reduction of RV/LV ratio in TTE and CT. CT analysis tend to show a higher reduction in RV/LV ratio

In 32 patients, both pre- and postprocedural transthoracic echocardiography was available, and RV/LV ratio change could be assessed (Fig. 1). In 31 (97%) cases, the RV/LV ratio improved and in 1 (3%) the ratio deteriorated. The median RV/LV ratio decreased from 1.19 (1.02–1.35) to 0.89 (0.78–1.00) by 24% (– 0.29, 95% CI – 0.3551 to – 0.2194, *p* < 0.001). An expected reduction within 24 h with anticoagulation only was previously described as 0.03 (2.5%) (Fig. 2A) [9]. A higher pre-RV/LV ratio correlated with a higher reduction (*R*^2^ = 0.57; *p* < 0.001; Fig. 2C). The RV/LV ratio reduction was similar between intermediate–high- and high-risk patients. Preprocedural pulmonary arterial pressure showed no significant correlation with RV/LV improvement (*R*^2^ = 0.063, *p* = 0.33), indicating that it is no reliable predictor of treatment response.

The RV/LV ratio change in CT was assessed in 16 patients. It was reduced from 1.33 (1.19–1.67) to 1.04 (0.87–1.25) by 32% (– 0.320; 95% CI – 0.4574 to – 0.1826, *p* < 0.001, Fig. 2B). CT estimated higher RV/LV ratios than TTE, as shown in the Blant–Altman plot and expressed by a mean difference of – 0.11 (Fig. 2D).

### Changes in physiological parameters

**Fig. 3:**
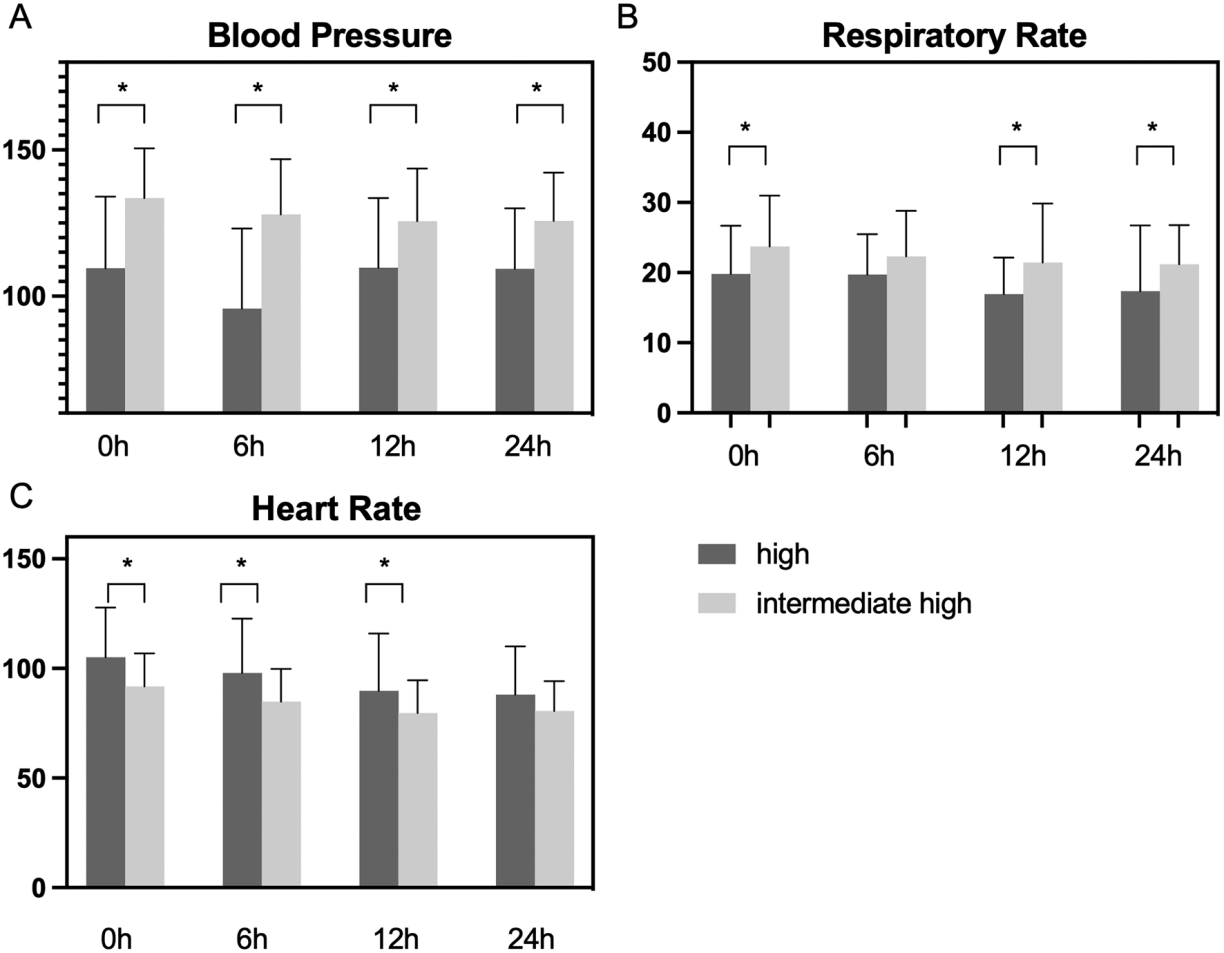
Changes in physiological parameters. **A** Significant differences in blood pressure between the risk groups were observed. Although both groups improved, significant differences remained after 24 h. **B** Significant differences in the respiratory rate between the risk groups were observed. No significant changes were observed after 24 h. **C** Significant differences in heart rate at the beginning equalized after 24 h and both groups improved significantly

Significant differences were observed in blood pressure, respiratory rate, and heart rate between high- and intermediate–high-risk patients (Fig. 3). High-risk patients had significantly lower blood pressure than intermediate–high-risk patients (110 vs. 134, 23.95 ± 4.53, 95% CI 14.9–32.9). It did not improve significantly within the groups, and significant differences persisted after 24 h (109.3 vs. 125.7, 16.40 ± 4.49, 95% CI 7.46–25.33). Respiratory rate was significantly higher in the intermediate–high-risk patients (19.79 vs. 23.71, 3.92 ± 1.68, 95% CI 0.59–7.26). No significant changes within the risk group were observed and differences between groups remained after 24 h (23.71 vs. 21.18, – 2.534 ± 1.210, 95% CI – 4.93 to – 0.14). However, significant differences in heart rate at the beginning (105 vs. 92, – 13.35 ± 3.96, 95% CI – 21.21 to – 5.5) were observed. After 24 h, these differences equalized (88 vs. 81, – 7.4 ± 4, 95% CI – 15.44 to 0.68, *p* = 0.07), with a significant improvement in both groups.

### Secondary outcomes

**Fig. 4:**
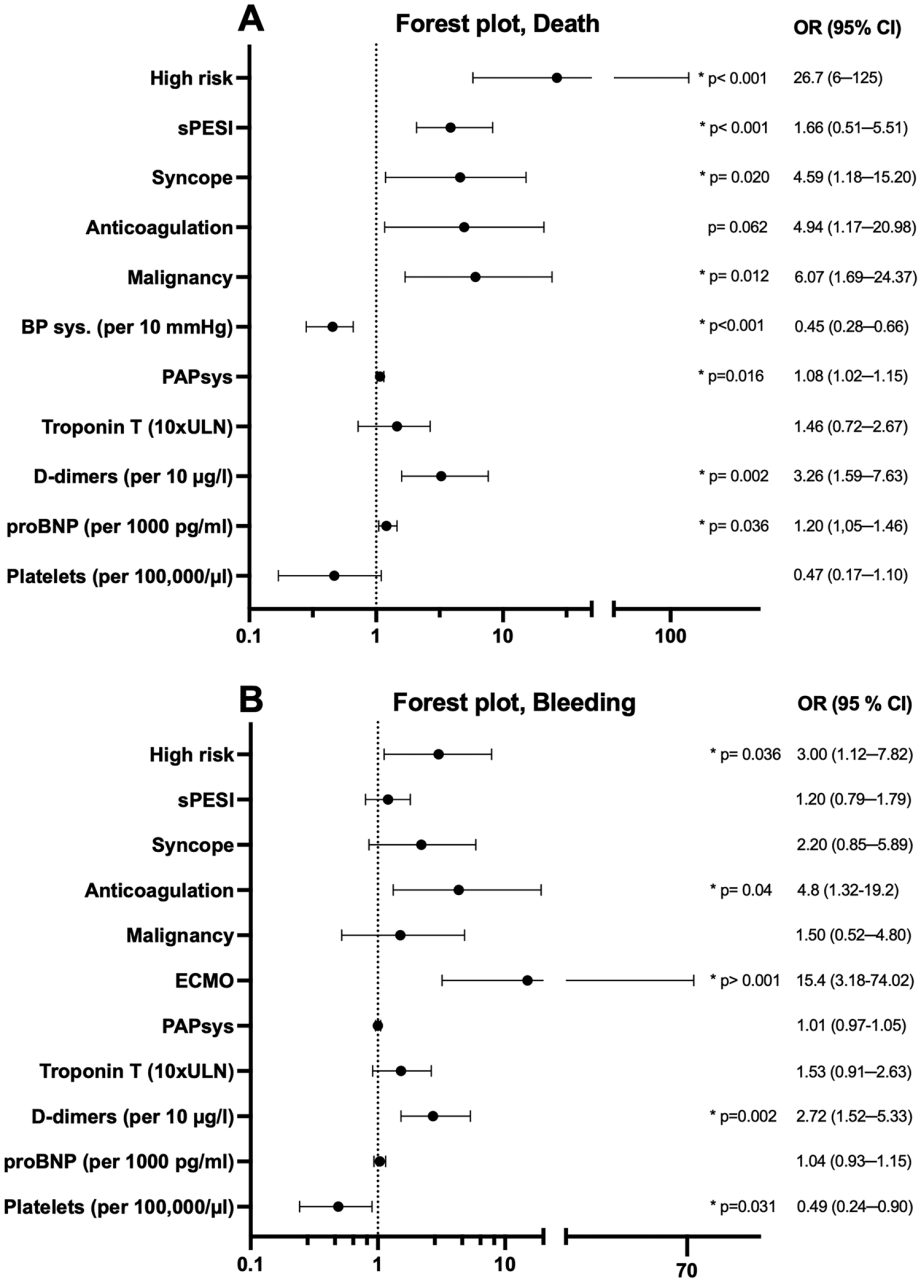
Associated risk factors of intrahospital death and bleeding. **A** Forest plot comparing odds ratios of clinical risk factors for intrahospital death. High risk, PAPsys, Syncope, Anticoagulation, malignancy, sPESI score, blood pressure, d-dimers and proBNP indicated a higher risk for intrahospital death. **B** Forest plot comparing odds ratios of clinical risk factors for bleeding. High risk, ECMO, anticoagulation, elevated D-dimer levels and low platelet count indicated an elevated risk for bleeding. Other clinical aspects did not correlate significantly

12 (11%) patients died, with a median of 119.5 (74─204.25) h from the time of intervention (S2 Supplementary Data). Figure 4A shows associated risk factors for intrahospital death. Patients who died had a higher initial sPESI (3 vs 1.4, *p* < 0.001) and nine (75%) received catecholamine therapy. High-risk stratification strongly correlated with death (OR 26.67, 95% CI 5.885–125.4), with 10 out of 12 deaths (83%) categorized as high-risk. The high-risk group had a mortality rate of 40%, while the intermediate–high-risk group only had 2.5%. Initial invasive systolic PAP correlated with death (OR 1.075, 95% CI 1.020–1.154, *p* = 0.016), whereas bleeding did not (OR 1.009, 95% CI 0.9660–1.052, *p* = 0.68). A significant correlation was found between malignancy condition (OR 6.07, 95% CI 1.69─24.37, *p* = 0.012), syncope at presentation (OR 4.59, 95% CI 1.18─15.20, *p* = 0.020), elevated proBNP per 1000 pg/ml (OR 1.20, 95% CI 1.05─1.46, *p* = 0.036), elevated D-dimers per 10 µg/ml (OR 3.26, 95% CI 1.59─7.63, *p* = 0.002), and low systolic blood pressure per 10 mmHg (OR 0.45, 95% CI 0.28─0.66, *p* = 0.002). In 28 (26%) patients, at least one postprocedural bleeding event occurred. Most of the patients experienced bleeding events within the first 24 h, with a median occurrence after 8 (6─12) h. 19 (18%) of the bleeding events occurred under running local lysis, representing 2/3 of all bleeding events. 14 were minor and 14 were major bleedings, according to the ISTH bleeding categorization. 11 (10%) major bleeding events occurred under running lysis. There were no fatal bleeding events. 15 (14%) patients required blood transfusions; however, no one required interventional hemorrhage control. Additionally, ten (9%) of them had an extracorporeal procedure. 18 (17%) patients were on antiplatelet therapy, 17 (16%) on aspirin, and 1 (1%) on clopidogrel. None of them were under dual antiplatelet therapy. Therapeutic anticoagulation was found in nine (8%) patients. Four (4%) received rivaroxaban, one (1%) received apixaban, and four (4%) received low molecular weight heparin. Antiplatelet therapy did not show any association with elevated bleeding events (OR 0.51, 95% CI 0.1479–1.757, *p* = 0.39). However, an association between therapeutic anticoagulation and bleeding events could be observed (OR 4.8, 95% CI 1.32–19.20, *p* = 0.04). Bleeding events correlated strongly with the use of ECMO (OR 15.4, 95% CI 3.178–74.02, *p* < 0.001. Also, high-risk stratification correlated with bleeding events (OR 3, 95% CI 1.116–7.815, *p* = 0.03) with a rate of 44% in the high-risk and only 17% in the intermediate–high-risk group. The rate of major bleedings was 36% in the high-risk and 6.2% in the intermediate–high-risk group, respectively. 5 of 14 major bleedings occurred at puncture sites. 3 of 14 events were classified as increased postoperative major bleedings. There were no documented intracranial bleedings.

Figure 4 shows associated risk factors. Significant correlations were found in platelet count per 100 000/µl (*p* = 0.031) and elevated D-dimer level 10 mg/l (*p* = 0.001) (Fig. 4B). sPESI score of patients with bleeding did not differ significantly from the overall population (1.6 vs 1.8; *p* = 0.38), and 17 patients (61%) were at intermediate–high risk.

A 6-month follow-up was available for 28 patients after a median time of 6.1 (5.2–6.4) months. Of those, six (21%) reported persistent dyspnea and seven (25%) reported reduced exercise capacity, but only in two patients (7%) signs of right ventricular dysfunction were found. There was no additional improvement in the RV/LV ratio compared to the postprocedural data. Two (7%) patients stopped their oral anticoagulation prematurely. Medication was rivaroxaban in 22 (79%) cases, apixaban in 3 (11%) cases, and edoxaban in 1 (4%) case. 19 (68%) qualified for prolonged oral anticoagulation because of elevated thromboembolism recurrence risk. 13 (46%) qualified for a reduced dose in extended anticoagulation.

## Discussion

This study found that USAT is a promising treatment option with high procedural success for patients with intermediate high-risk PE. Under USAT treatment, the RV/LV ratio improved significantly, especially in those with a higher RV/LV ratio at presentation. Syncope, d-dimers, and proBNP at presentation, but not troponin, indicated increased risk of intrahospital death. High d-dimers and low platelets indicated a risk of bleeding. These predictors might help identify patients at higher risk for death or bleeding among this heterogenous group. More than half of the patients who presented for structured follow-up in an outpatient clinic had an indication for OAC beyond 6 months.

Baseline characteristics and comorbidities of the study cohort were similar to those of other studies, reported by Piazza et al. and Klein et al. [10, 11]. Demographic analysis revealed slightly higher body weight in North American cohorts compared to European cohorts. Technical success rates in this cohort were high, with only rare cases of unsuccessful catheter placement, as reported elsewhere. Klein et al. described a smaller number of bilateral catheters (16% vs. 88%) resulting in a lower dose of lysis (11.5mg vs. 20mg). Unlike Klein et al., we could not report any periprocedural device failures [10]. We observed a reduction of RV/LV ratio of 0.29 (24%). Engelhardt et al. described a reduction from 1.33 ± 0.24 to 1.00 ± 0.13 (*p* < 0.001) [12], Piazza et al. reported a reduction from 1.55 to 1.16 (*p* < 0.0001), and similarly Becattini reported a reduction from 1.36 to 1.03 [11, 13]. A higher initial RV/LV ratio was associated with a greater magnitude of reduction (*R*^2^ = 0.57; *p* < 0.001). This aligns with Kucher et al.’s study, which described an early reduction by 0.29 in the USAT group. Simultaneously in the heparin group, a reduction only by 0.03 was observed within 24 h. After 90 days, a trend in favor of the USAT group remained, but there were no more significant differences [9].

Other studies reported lower mortality rates: 2.7% (Piazza et al.), 3.9% (Klein et al.), and Engelhardt describing a rate of 0% [10–12]. In this study, the overall mortality rate was 11% among 107 patients, with this higher rate largely attributable to the greater proportion of high-risk patients. 40% of high-risk patients died, whereas only 2.5% of intermediate–high-risk patients died. In contrast, other recent cohort studies, such as reported by Sterling et al., had a lot lower percentages of high-risk patients (5% vs. 23%) resulting in lower mortality rates (1% vs 11%) [14]. Syncope, d-dimers, and proBNP could be identified as relevant risk factors associated with higher mortality rates. Other studies reported no associated risk factors.

These findings show a 13% rate of major bleeding events, which is slightly higher compared to other studies, at 11.7% (Klein et al.) and at 10% (Piazza et al.) [10, 11]. Klein et al. used a lower overall dose of rtPA (11.5 mg vs 20 mg). SEATTLE-II was a prospective study with a highly selected cohort, excluding patients with high bleeding risks. Higher rates of bleeding events were strongly associated with high-risk patients, especially those on ECMO (80% on ECMO vs. 36% in the high-risk vs. 6.2% in the intermediate–high-risk group) Furthermore, patients were slightly younger than the patients in this cohort (59 vs 64 years). Age, kidney function, or operations were no exclusion criteria, leading to a cohort with a higher burden of disease and risk for complications in this cohort. Sterling et al. reported a lower rate of major bleeding events (1.6% vs 13%) [14]. Low platelets and high d-dimer levels correlated significantly with a higher bleeding risk. At follow-up no case of CTEPH was observed, correlating well with other studies that report very low numbers of CTEPH at follow-up [10].

These findings suggest that a well-defined selection of patients undergoing this procedure is essential. Per guidelines, systemic lysis is the first-line recommendation for high-risk pulmonary embolism [3]. At the time of data acquisition, USAT was the only catheter-based system used at this site. Robust randomized data for long-term clinical outcomes are still missing [15]. The absence of alternative catheter-directed methods by that time may be an explanation for the relatively high percentage of patients at risk for adverse events. Other publications indicate that as soon as large-bore thrombectomy (LBT) is available, there is a shift from CDT-primary treatment modality to an LBT-primary treatment modality [16]. In both retrospective and prospective analyses, there were no significant differences in mortality or bleeding rates [16, 17]. However, in a win-ratio comparison there were significantly fewer episodes of clinical deterioration in the LBT group [17]. USAT was used in intermediate low-risk patients in this cohort according to individual treatment decision by the treating physicians. These findings underline both the real-world challenges of implementing a new treatment method and the urge for a well-defined evidence-based patient selection. Therefore, ongoing prospective studies such as HI-PEITHO (NCT04790370) and PEERLESS II (NCT06055920) are expected to clarify recommendations for this vulnerable, yet heterogeneous patient cohort.

## Strengths and limitations

In this retrospective single-center cohort study, the decision to perform USAT was made by the treating physician to the best of their knowledge, but without properly defined criteria or randomization, leading to a highly selected cohort. This selection bias may prohibit an unrestricted generalization of the findings to other PE populations. However, these data provide a valuable contribution in terms of real-world use of USAT. Requiring rapid decision-making, the emergency setting of the condition often results in a loss of data and information along the treatment pathway. Although a right ventricular dysfunction was described in the patients’ records, not all raw image data of transthoracic echocardiography were available for analysis, limiting statistical analysis. Furthermore, some patients were transferred from other hospitals for treatment, which also contributed to a loss of information. The small number of follow-up patients also impairs data quality. Beyond scientific questions, these findings may help in optimizing clinical workflows to increase data quality, resulting in an overall improvement of treatment quality. These insights can be easily transferred to other disciplines and aspects.

## Conclusion

In this single-center study, we observed high technical success for USAT in patients with pulmonary embolism. Significant differences in safety outcomes between intermediate–high and high risk highlight the importance of thorough patient selection. Syncope, d-dimers, and proBNP at presentation, but not troponin, indicated increased risk of intrahospital death. High d-dimers and low platelets indicated a risk of bleeding. The RV/LV ratio improved in the majority of patients with available follow-up imaging with a more pronounced improvement in those with higher RV/LV ratio at presentation.

## Supplementary Information

Below is the link to the electronic supplementary material.

Supplementary file1 (DOCX 78 KB)

## Data Availability

The datasets used and/or analyzed during the current study are available from the corresponding author upon reasonable request.

## References

[1] Wendelboe AM, Raskob GE (2016) Global burden of thrombosis: epidemiologic aspects. Circ Res 118(9):1340–1347 27126645 10.1161/CIRCRESAHA.115.306841

[2] Jiménez D et al (2018) Management appropriateness and outcomes of patients with acute pulmonary embolism. Eur Respir J 51(5):1800445 29724918 10.1183/13993003.00445-2018

[3] Konstantinides SV et al (2020) 2019 ESC Guidelines for the diagnosis and management of acute pulmonary embolism developed in collaboration with the European Respiratory Society (ERS). Eur Heart J 41(4):543–603 31504429 10.1093/eurheartj/ehz405

[4] Gayou EL et al (2019) Nationwide trends in use of catheter-directed therapy for treatment of pulmonary embolism in Medicare beneficiaries from 2004 to 2016. J Vasc Interv Radiol 30(6):801–806 31040058 10.1016/j.jvir.2019.02.024

[5] Pruszczyk P et al (2014) Prognostic value of echocardiography in normotensive patients with acute pulmonary embolism. JACC Cardiovasc Imaging 7(6):553–560 24412192 10.1016/j.jcmg.2013.11.004

[6] Frémont B et al (2008) Prognostic value of echocardiographic right/left ventricular end-diastolic diameter ratio in patients with acute pulmonary embolism: results from a monocenter registry of 1416 patients. Chest 133(2):358–362 17951624 10.1378/chest.07-1231

[7] Zaidi A et al (2020) Echocardiographic assessment of the right heart in adults: a practical guideline from the British Society of Echocardiography. Echo Res Pract 7(1):G19-g41 32105053 10.1530/ERP-19-0051 PMC7077526

[8] Schulman S, Kearon C (2005) Definition of major bleeding in clinical investigations of antihemostatic medicinal products in non-surgical patients. J Thromb Haemost 3(4):692–694 15842354 10.1111/j.1538-7836.2005.01204.x

[9] Kucher N et al (2014) Randomized, controlled trial of ultrasound-assisted catheter-directed thrombolysis for acute intermediate-risk pulmonary embolism. Circulation 129(4):479–486 24226805 10.1161/CIRCULATIONAHA.113.005544

[10] Klein F et al (2022) EKOS^™^ Jena experience: safety, feasibility, and midterm outcomes of percutaneous ultrasound-assisted catheter-directed thrombolysis in patients with intermediate-high-risk or high-risk pulmonary embolism. Can Respir J 2022:7135958 35265230 10.1155/2022/7135958 PMC8898866

[11] Piazza G et al (2015) A prospective, single-arm, multicenter trial of ultrasound-facilitated, catheter-directed, low-dose fibrinolysis for acute massive and submassive pulmonary embolism: the SEATTLE II study. JACC Cardiovasc Interv 8(10):1382–1392 26315743 10.1016/j.jcin.2015.04.020

[12] Engelhardt TC et al (2011) Catheter-directed ultrasound-accelerated thrombolysis for the treatment of acute pulmonary embolism. Thromb Res 128(2):149–154 21641020 10.1016/j.thromres.2011.05.014

[13] Becattini C et al (2010) Bolus tenecteplase for right ventricle dysfunction in hemodynamically stable patients with pulmonary embolism. Thromb Res 125(3):e82–e86 19833379 10.1016/j.thromres.2009.09.017

[14] Sterling KM et al (2024) Prospective multicenter international registry of ultrasound-facilitated catheter-directed thrombolysis in intermediate-high and high-risk pulmonary embolism (KNOCOUT PE). Circ Cardiovasc Interv 17(3):e013448 38264938 10.1161/CIRCINTERVENTIONS.123.013448 PMC10942169

[15] Pruszczyk P et al (2022) Percutaneous treatment options for acute pulmonary embolism: a clinical consensus statement by the ESC Working Group on Pulmonary Circulation and Right Ventricular Function and the European Association of Percutaneous Cardiovascular Interventions. EuroIntervention 18(8):e623–e638 36112184 10.4244/EIJ-D-22-00246 PMC10241264

[16] Feroze R et al (2023) Comparison of large-bore thrombectomy with catheter-directed thrombolysis for the treatment of pulmonary embolism. J Soc Cardiovasc Angiogr Interv 2(1):100453 39132536 10.1016/j.jscai.2022.100453 PMC11308115

[17] Jaber WA et al (2025) Large-bore mechanical thrombectomy versus catheter-directed thrombolysis in the management of intermediate-risk pulmonary embolism: primary results of the PEERLESS randomized controlled trial. Circulation 151(5):260–273 39470698 10.1161/CIRCULATIONAHA.124.072364 PMC11789609

