# Supplementary material for: Characterizing technical success and clinical outcomes in patients with pulmonary embolism treated with ultrasound-assisted catheter-directed thrombolysis (USAT): a retrospective, single-center cohort study
Source: Clin Res Cardiol. 2025 May 19;115(3):449–58. doi: 10.1007/s00392-025-02643-2 (PMC12894431; doi:10.1007/s00392-025-02643-2)
Supplement: Supplementary file 1 — Supplementary file1 (DOCX 78 KB) [file 392_2025_2643_MOESM1_ESM.docx]

Appendix

S1

| Inclusion criteria | EKOS procedure between May 2019 and June 2022 |
| --- | --- |
| Exclusion criteria | EKOS used in non PE Patients  Significant deviation from standard procedure |

S2 . Kaplan Meier Curves of Survival and Bleeding
